# 12.2 METABOLIC CONSEQUENCES OF DEVELOPMENTAL NMDA RECEPTOR HYPOFUNCTION

**DOI:** 10.1093/schbul/sby014.045

**Published:** 2018-04-01

**Authors:** Adam Funk, Catharine Mielnik, Sinead O’Donovan, Courtney Sullivan, Yuxiao Chen, Robert McCullumsmith, Amy Ramsey

**Affiliations:** 1 University of Cincinnati; 2 University of Toronto

## Abstract

**Background:**

Several imaging and postmortem studies provide evidence that, in the brains of people with schizophrenia, there are alterations in glucose metabolism and energy utilization. However, it is difficult to determine whether altered excitatory transmission alters bioenergetics that then contributes to symptoms of the disorder. We have used a mouse model to begin to address these questions. GluN1 knockdown mice have a mutation that reduces NMDA receptor levels throughout development and maturity.

**Methods:**

We affinity purified PSD95 protein complexes from GluN1KD and WT brains (n=3 per group) and ran each sample through our liquid chromatography tandem mass spectrometry (LC-MS/MS) protocol in singlicate. We performed pathway analysis with the EnRICHr suite of bioinformatic tools and compared WT to GluN1KD PSD95 interactomes using the top 20 differentially expressed proteins. We also studied how NMDA receptor hypofunction changes the expression of genes related to glucose metabolism and bioenergetics by quantitative PCR of brain cDNA from WT and GluN1 knockdown mice.

**Results:**

Pathway analysis revealed that WT mice showed pathways relevant for synaptic plasticity (as expected), while GluN1KD analyses yielded proteins related to glucose metabolism and utilization. Gene expression analysis revealed that GluN1 knockdown mice have significant decreases in the expression of Slc16a3, Slc2a1, and Slc2a3, which are the genes for the monocarboxylate transporter (MCT4), and glucose transporters 1 and 3 (GLUT1 and GLUT3).

**Discussion:**

Our results show that NMDA receptor dysfunction leads to expression changes that would reduce glucose and lactate transport into neurons. The synaptic proteome of NMDAR deficient mice shows an increase in glycolytic enzymes located at the synapse. These data suggest a profound shift in the composition of the cortical excitatory synaptic proteome in GluN1KD mice, with apparent increases in neuroenergetic substrates in neurons. At the same time, there were significant decreases in the levels of transporters that bring glucose and the primary energy substrate, lactate, into neurons. The MCT4 shuttles lactate from astrocytes to neurons, which can then be used for oxidative respiration in neurons. GLUT1 is responsible for transport of glucose across the blood-brain-barrier, and GLUT3 is expressed on neurons and is responsible for glucose uptake in those cells. Notably, we have identified that these transporter gene transcripts are reduced in postmortem brains of people with schizophrenia. Thus, this mouse may be a useful tool to model bioenergetic changes that are observed in schizophrenia, and study functional outcomes when glucose metabolism is improved.

